# Biliary Obstruction due to a Pancreatic Plasmacytoma

**DOI:** 10.1155/2018/9017617

**Published:** 2018-09-04

**Authors:** Min Ho Cho, Rohan Mandaliya, Jennifer Tran, Wen Lee, Mitesh Patel

**Affiliations:** ^1^Department of Medicine, MedStar Washington Hospital Center, Washington, DC, USA; ^2^Division of Gastroenterology and Hepatology, MedStar Georgetown University Hospital, Washington, DC, USA; ^3^Department of Pathology, MedStar Washington Hospital Center, Washington, DC, USA; ^4^Division of Gastroenterology, MedStar Washington Hospital Center, Washington, DC, USA

## Abstract

Obstructive jaundice, weight loss, and anorexia often raise a concern for pancreatic malignancy. Although pancreatic adenocarcinoma is the most common form of pancreatic cancer, not all pancreatic malignancies are exocrine in origin. With advancement in endoscopic ultrasound with fine needle aspiration, it has become easier to make correct diagnosis. Plasmacytoma of pancreas is a solitary tumor of plasma cells and it can also lead to the same clinical presentations. Immunohistochemistry is required to make the diagnosis of plasmacytoma. However, when there are other systemic manifestations, such as hypercalcemia, renal injury, and anemia, a diagnosis of multiple myeloma should be suspected and confirmed by cytology or biopsy. It is very important to differentiate plasmacytoma from multiple myeloma as the management for each is different. Herein, we describe a case of multiple myeloma presenting as a pancreatic plasmacytoma causing obstructive jaundice.

## 1. Background

About 60 to 70% of pancreatic adenocarcinomas are located in the head of pancreas, causing obstructive jaundice from the compression of the common bile duct [1]. Although adenocarcinoma is most common, not all pancreatic tumors are endocrine or exocrine in nature. Some of these uncommon tumors include metastases, pseudopapillary neoplasm, and hematologic malignancy, such as lymphoma. Even rare is a pancreatic plasmacytoma.

Plasmacytoma is a solitary tumor of plasma cells. Since the first case report of pancreatic plasmacytoma by Hefferman published in 1947 [2], there have been about 63 English literatures published [3]. Plasmacytoma of pancreas often raises a concern for adenocarcinoma as their clinical presentations are similar, such as, abdominal pain, weight loss, asthenia, and anorexia. However, the pathology, prognosis, and management of pancreatic plasmacytoma are unique.

Herein, we describe a case of multiple myeloma presenting as a pancreatic plasmacytoma causing obstructive jaundice.

## 2. Objective

The objective is to describe a case of an aggressive pancreatic plasmacytoma.

## 3. Case Report

**Figure 1:**
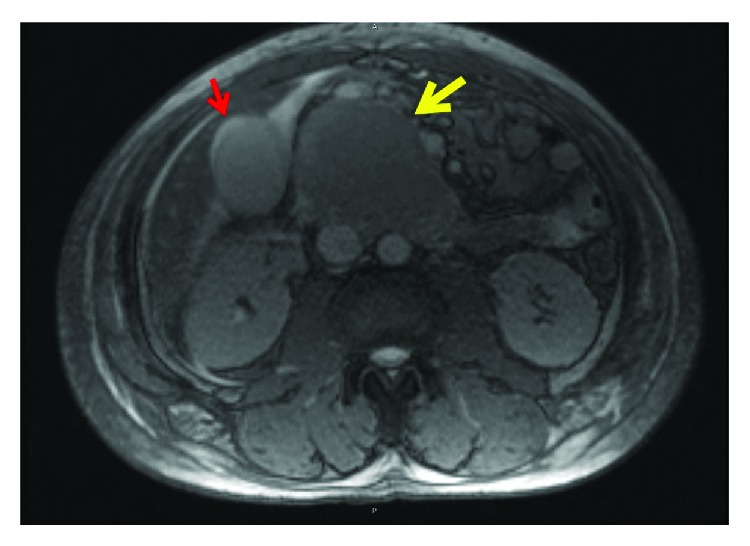
MRI of abdomen T2 sequence showed a moderately T2 hyperintense, round, well-circumscribed homogeneous mass, measuring approximately 6.3 × 5.9 × 8.2 cm, arising off the head of the pancreas (yellow arrow). There was also a mild thickening of gallbladder wall (red arrow).

**Figure 2:**
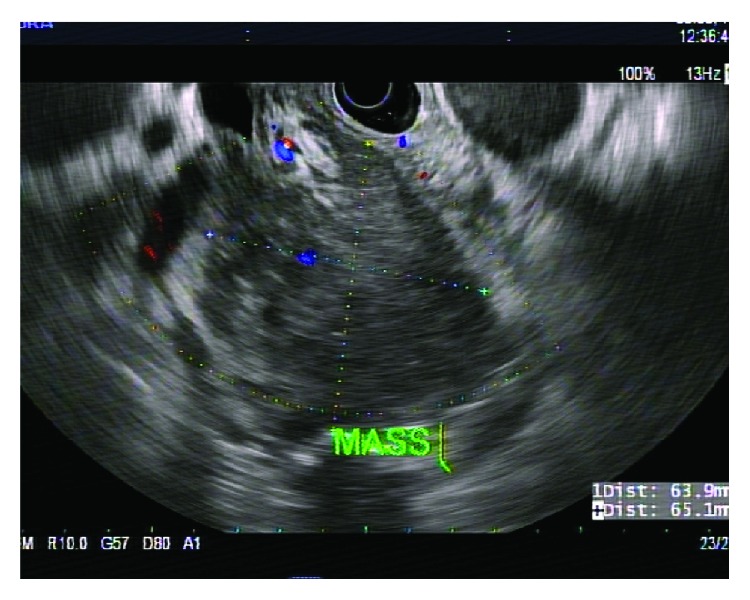
Endosonographic image demonstrating a well-defined round, hypoechoic mass, measuring 65 mm × 64 mm in maximal cross-sectional diameter.

**Figure 3:**
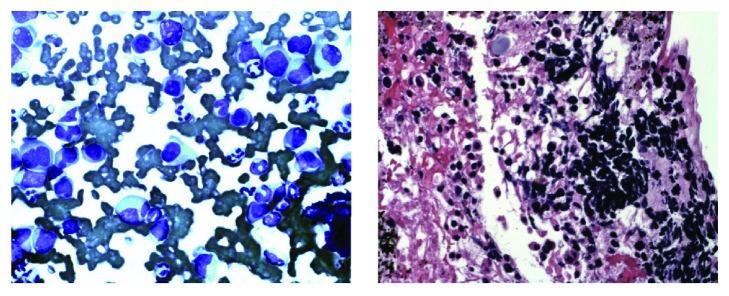
Cytology smear (Diff-Quick stain) of the FNA sample on the left showed numerous plasma cells with atypical nucleoli under 400x magnification. Hematoxylin and eosin staining of the FNA sample showed abundant lymphocytes under 400x magnification.

**Figure 4:**
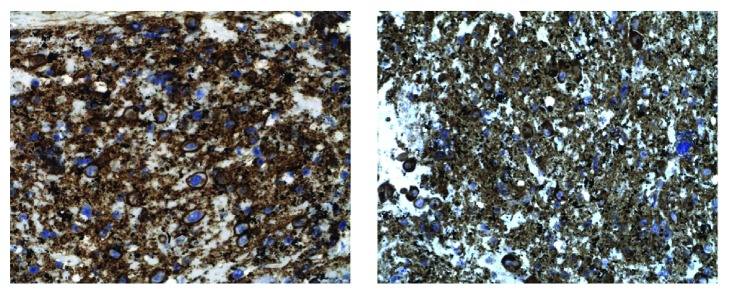
Immunohistochemistry of the FNA sample showed positive staining for CD138 under 400x magnification on the left side and positive staining for Lambda light chain under 400x magnification on the right side.

A 60-year-old African-American male presented with worsening abdominal pain and weight loss of 30 pounds in one month. Physical examination revealed scleral icterus with mild abdominal tenderness. Laboratory results showed anemia (hemoglobin of 5.7 gm/dL and hematocrit 16.6%), renal failure (creatinine of 20.89 mg/dL), hypercalcemia of 11.3 mg/dL, lipase of 8039 unit/L, alkaline phosphatase of 534 unit/L, and total bilirubin of 17.4 mg/dL. MRI of the abdomen showed a well-circumscribed homogenous mass at the head of pancreas obstructing the biliary system (Figure 1). EUS showed a hypoechoic mass (Figure 2) and smear of the FNA sampling with a 22G needle revealed numerous atypical plasma cells displaying increased cell size, fine nuclear chromatin, and prominent nucleoli (Figure 3). Hematoxylin and eosin stain showed basophilic stained plasma cells (Figure 3). Given the extramedullary plasmacytoma, anemia, renal failure, and hypercalcemia, a diagnosis of multiple myeloma was suspected and confirmed with cytology and bone marrow biopsy with immunohistochemistry. Immunohistochemistry was positive for CD138 and IgA Lambda consistent with plasmacytoma (Figure 4).

Patient was treated with radiation and chemotherapy for the pancreatic plasmacytoma and multiple myeloma, respectively. His multiple myeloma did not respond to chemotherapy. Unfortunately, the patient developed further complications, including malignant ascites and pericardial effusion. The disease was refractory to chemotherapy and he passed away 10 months after the time of the diagnosis.

## 4. Discussion

Multiple myeloma is clonal proliferation of plasma cells. About 5% of plasma cell tumors form an isolated solitary lesion, called plasmacytoma [4]. It can occur either inside (medullary plasmacytoma) or outside the bone marrow (extramedullary plasmacytoma), although the incidence of the former is approximately 40% higher [4].

The upper respiratory tract is the most common predilection site for extramedullary plasmacytoma. However, approximately 10% of it arises from gastrointestinal tract with the stomach being the most frequently involved organ. There are only about 63 case reports of pancreatic plasmacytoma, including both primary and secondary lesions. Secondary plasmacytoma is more common with only several cases of primary pancreatic plasmacytoma [5].

Common signs and symptoms in patients with pancreatic plasmacytoma included abdominal pain, obstructive jaundice, and anorexia, which were also seen in our patient [3]. These symptoms raise a concern for a pancreatic adenocarcinoma, especially when there is biliary obstruction as well.

It is very important to make a correct diagnosis as the management of adenocarcinoma of pancreas is different from pancreatic plasmacytoma. With EUS and FNA sampling, it has become easier to make the diagnosis. Diagnosis of plasmacytoma entails histopathology and immunohistochemistry which will show abundant plasma cells and either Kappa or Lambda dominant light chain stain, respectively.

It is also very imperative to make a distinction between primary and secondary plasmacytoma as the prognosis and management are different. Management of isolated primary plasmacytoma of pancreas involves radiation therapy and it has a good prognosis. However, management of secondary plasmacytoma requires systemic steroids and chemotherapy. Patients with secondary plasmacytoma have worse prognosis with higher recurrence rate [6]. Therefore, once plasmacytoma is identified, subsequent investigation should include bone marrow biopsy as well as radiography, either PET/CT or MRI, to rule out multiple myeloma [7].

In conclusion, this report describes a rare case of pancreatic plasmacytoma, resulting in abdominal pain and obstructive. Our patient most likely had secondary plasmacytoma although the timeline of disease progression is not completely delineated. The rate of disease progression seen in our patient is unusual but confers the idea that patients with an extramedullary disease have a poorer prognosis than those with multiple myeloma alone.
